# Femtosecond Laser Treatment of Copper Current Collectors and Their Application in Li-Ion Batteries

**DOI:** 10.1021/acsaenm.5c00589

**Published:** 2025-09-16

**Authors:** Maciej Ratynski, Michal Krajewski, Tomas Tamulevičius, Yaroslav Vasyliovych Bobytskyy, Joanna B. Kisała, Piotr Krzeminski, Bartosz Hamankiewicz, Andrzej Czerwinski

**Affiliations:** † Faculty of Chemistry, 49605 University of Warsaw, Pasteura 1, 02-093 Warsaw, Poland; ‡ Institute of Materials Science, 70309 Kaunas University of Technology, K. Baršausko St. 59, 51423 Kaunas, Lithuania; § Faculty of Science and Technology, 49726 University of Rzeszow, Pigonia 1, 35-310 Rzeszow, Poland

**Keywords:** laser treatments, current collectors, silicon, lithium-ion batteries, negative electrodes

## Abstract

Current collectors (CCs) play an important role in enhancing the electrochemical performance of lithium-ion batteries (LiB). Research shows that increasing the surface roughness of copper foil helps improve the bonding strength between the current collector and the active material, reduces the contact resistance between them, and consequently enhances the battery’s rate discharge performance and cycling stability. In the present work, a copper current collector modification by femtosecond laser treatment forming quasiperiodical nanostructures was proposed. The modified current collectors were examined through scanning electron microscopy (SEM) imaging and roughness measurements and further tested with silicon particles in Li-ion cells through galvanostatic charge/discharge experiments. As a result of the laser process, one can observe increasing contact between the current collector and the active material grains, which creates a buffer zone for the volume expansion of the active material during charging and discharging. The results show increased specific capacities, cyclabilities, and Coulombic efficiencies for modified current collectors compared with standard copper foil. It was demonstrated that implementing laser treatment into standard procedures of electrode manufacturing for lithium-ion batteries can easily improve the electrochemical performance of active materials, especially those that experience large volume changes during cycling.

## Graphical abstract

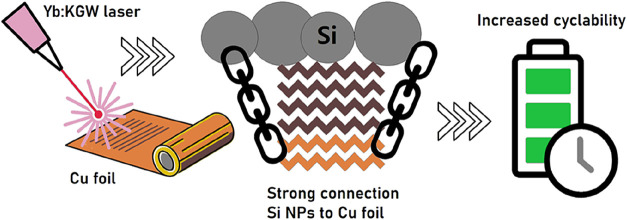

## 1 Introduction

Lithium-ion batteries represent the most widely employed electrochemical energy storage systems at present. The increasing demand for higher energy density and the development of lighter, more compact battery architectures continuously propel advancements in both active material selection and fabrication methodologies. A conventional lithium-ion battery is composed of a cathode, an anode, a separator, and an electrolyte. Recently, significant attention has been directed toward alloy-type compounds—such as tin, germanium, aluminum, and silicon—as promising candidates for anode-active materials.^1–4^ These elements exhibit significantly higher theoretical specific capacities compared with conventional graphite-based anodes (372 mAh g^–1^).^5^ The Sn, Al, Ge, and Si theoretical specific capacity is equal to 993, 994, 1600, and 3590 mAh g^–1^, respectively.^4^ Among them, silicon has emerged as a particularly attractive candidate for next-generation anode materials owing to its exceptionally high capacity, natural abundance, and relatively low cost. However, alloy-type anode materials, including silicon, are inherently limited by substantial volume changes that occur during the lithiation and delithiation processes. In the case of silicon, the volumetric expansion can reach up to approximately 300%.^6^ Such dramatic volume fluctuations induce severe mechanical stresses within the electrode, often resulting in structural degradation. This includes the pulverization of the active material, interparticle disconnection, and delamination of the electrode film from the current collector. These effects lead to loss of electrical contact and active material isolation, further amplified by the repeated formation and breakdown of the solid electrolyte interphase (SEI) layer, which forms rapidly during cycling.^7^

Despite significant research efforts,^8,9^ the formation mechanism of the SEI layer is still not fully understood. In carbonate-based liquid electrolytes, it typically exhibits a multilayered structure.^10^ The inner inorganic layer consists mainly of lithium salts such as LiF, Li_2_O, and Li_2_CO_3_, while the outer organic layer comprises monomers and oligomers formed from electrolyte decomposition.^11^ Due to ongoing degradation and reformation, SEI composition evolves with the electrode potential. Initially, porous LiF forms and is gradually surrounded by organic products. Ultimately, a compact inorganic layer (LiF and others) forms near the electrode surface, overlaid by a porous mixture of LiF and organic species.^12^ SEI thickness may vary from 40 to 240 nm, depending on cell operation and surface characteristics.^13^

At the silicon interface, the SEI includes Li_2_O and Li_*x*_SiO_*y*_, formed from lithium reacting with the native oxide layer^14^ alongside other reduced species. Different materials may be covered by surface oxides that can be easily reduced to Li_2_O at low potentials. In case of metallic three-dimensional (3D) current collectors, the reduction of those may alter the SEI layer properties and result in variation of the whole cell stability.

SEI composition is strongly influenced by the electrochemical history of the silicon electrode, particularly the rate and method of potential or current application, as well as spatial current distribution.^15^ It is worth noticing that evenly distributed current density, accomplished by 3D current collectors, may be a significant factor affecting the cycle life of the battery as it will promote the formation of even and potentially stronger SEI layers.

Given the SEI instability and mechanical fragility, electrolyte additives play a key role in improving the cycling performance of silicon anodes. To accommodate silicon’s volume changes, the SEI should be elastic and preferably consist of polymeric or oligomeric compounds. Suitable additives, reduced at higher potentials (compared with pure EC:DMC electrolyte), are promoting the formation of a more stable SEI. Widely used additives include vinylene carbonate (VC), lithium bis­(oxalato)­borate (LiBOB), lithium difluoro­(oxalato)­borate (LiDFBOB), and fluoroethylene carbonate (FEC).^11^ FEC leads to a thinner, less porous SEI compared with that formed in pure EC. X-ray photoelectron spectroscopy (XPS) studies indicate increased levels of inorganic fluorinated species (e.g., LiF, SiF_*x*_) in the SEI formed with FEC additive. Decomposition of FEC and VC likely produces insoluble polymeric species, enhancing SEI flexibility and mechanical robustness during cycling. Several approaches have been developed to alleviate the adverse effects of silicon’s volume changes. One effective strategy involves the use of engineered nanostructures, such as nanopillars, thin films, nanowires, and carbon–silicon hollow spheres.^16–20^ These designs improve cycling stability by better accommodating volume fluctuations. However, their high surface area often leads to poor first-cycle Coulombic efficiency (due to a high surface area) and increased electrolyte decomposition. Additionally, the complex and costly fabrication processes limit their scalability and suitability for industrial applications. As a result, while nanostructuring offers valuable performance benefits, its practical implementation remains a significant challenge. To achieve a high energy density and prolonged cycle life, strong adhesion between the active material and the metallic current collector is critical. Adequate interfacial bonding minimizes electrical resistance and prevents delamination caused by mechanical stresses during cell assembly or repeated volume expansion and contraction during cycling. An effective strategy to further enhance the durability of silicon-based electrodes involves replacing the conventional flat copper foil with a three-dimensional (3D) current collector. The increased surface area and mechanical interlocking provided by 3D architectures improve adhesion, accommodate volume changes more effectively, and facilitate uniform current distribution.^21^ Currently, the research on 3D current collectors is focused on Cu, Ni, Ti, or carbon-based collectors. Copper collectors are widely used because of their high electrical conductivity and similarity to state-of-the-art, flat Cu collectors widely used in the Li-ion industry.^22^

Nickel and titanium are often used in the form of 3D foam with open pores^23–26^ or chemically etched thin foils.^27^ Chemically etched, rough Ni foil covered by a vacuum-deposited thin Si layer showed great cycling stability and retained ca. 1800 mAh g^–1^ after 200 cycles.^27^ 3D foams also presented great improvements, leading to a capacity of over 655 mAh g^–1^ after 1000 cycles.^23^

A similar modifications of the copper-based 3D current collectors, where multiple 3D foams,^28^ chemically modified,^29^ electrochemically etched,^30^ or electrochemically deposited rough foils, were successfully fabricated.^21^ Copper foam with an average pore size of approximately 50 μm, when impregnated with a conventional silicon-based slurry, demonstrated significantly reduced internal resistance and enhanced cycling performance compared with flat current collectors. This 3D architecture retained a specific capacity of 800 mAh g^–1^ after 60 cycles,^28^ highlighting its potential for improving electrode durability. However, the effective impregnation of such complex porous structures remains a major challenge for large-scale manufacturing. Ensuring uniform slurry distribution and strong adhesion within the 3D matrix is critical, and current techniques may lack the precision or throughput required for industrial implementation. Generally speaking, chemical modification of the thin foil is more likely to be implemented on an industrial scale due to similarities to the currently used process.

The slurry casting method is commonly employed for electrode fabrication, wherein a homogeneous mixture of active material, conductive additive, and polymer binder is uniformly applied to the surface of current collectors. However, the inherently smooth surface of commercial collector foils, such as copper, often leads to weak interfacial adhesion. This results in an elevated contact resistance and poor mechanical bonding between the active layer and the current collector, ultimately compromising the structural integrity of the electrode. These issues contribute to reduced electronic conductivity and accelerate performance degradation during repeated charge/discharge cycles.^31^ Therefore, the surface morphology of the current collector, along with the electrochemical contact and interfacial bonding strength between the collector and active materials, is widely recognized as a critical factor governing the cycling stability and overall performance of lithium-ion battery (LIB) cells. Careful control over current collector surface preparation and slurry casting is essential, as electrode stability is strongly influenced by the size and distribution of surface features, such as hills and valleys. Structures that are too large may fail to effectively anchor the majority of the active material, leaving much of it unsupported and prone to delamination. Conversely, features that are too small interact with only the bottom of the active layer, offering limited mechanical reinforcement. For instance, a rough copper foil with disproportionately large surface structures relative to the silicon layer thickness resulted in suboptimal performance—exhibiting a 50% capacity loss after 80 cycles, despite the silicon coating being only 0.5 μm thick.^32^

A more structured current collector, featuring surface cavities in the submicrometer range, was fabricated via thermal reduction of Cu­(OH)_2_ nanoneedles. This process yielded a rough copper substrate with cavity sizes on the order of several hundred nanometers. When coated with a silicon-based slurry containing 0.7 mg cm^–2^ of silicon, the resulting electrode achieved areal capacities comparable to those of its commercial counterparts.

Electrochemical evaluation using the capacity-limited discharge method demonstrated excellent cycling stability over 1000 cycles. This performance highlights the effectiveness of the nanoscale cavity design in accommodating silicon’s volume expansion, thereby maintaining structural integrity and stable electrochemical behavior throughout prolonged cycling.^33^ Other techniques such as selective etching also presented an improved adhesion strength between the active material layer and rough current collector surface and better battery cycle life when the size of the utilized microstructures was beneficial.^29,32^

Current trends in collector surface modification primarily aim to enhance adhesion between the metal substrate and the active material layer, as well as to improve the collector’s corrosion resistance. A variety of techniques have been explored to achieve these goals, including anodization and electrochemical etching processes,^34^ chemical etching with strong acidic or basic solutions,^35^ a coating treatment,^36^ ultrasonic peening technique, and orthogonal ploughing/extrusion (PE) machining.^37^ However, the implementation of such surface modification techniques often significantly increases production costs and may raise concerns regarding environmental compatibility. As a result, there is a growing demand for more efficient, cost-effective, and environmentally benign methods for structuring the surface of current collectors—ideally those that can be seamlessly integrated into large-scale manufacturing processes without compromising performance. LIPSS are parallel periodic lines formed in a self-assembled manner on the surface of the laser-irradiated material. In recent years, femtosecond lasers have gained considerable attention in the field of texturing current collectors due to their low thermal damage and high accuracy.^38^ Wang et al. developed a hierarchical micro/nanostructure current collector on a commercial Cu foil (12 μm thick) with a surface roughness of 485 nm. When applied to a graphite anode, the textured Cu foil showed a capacity retention of 74.7% after 200 cycles.^39^ In another study, Zhang et al. fabricated a 500 μm periodic circular pattern on the surface of a 10 μm thick Cu foil. Compared with the original electrode, the retained capacity increased by 30% after 100 cycles.^40^

Similar findings were reported by Wang et al., who employed femtosecond laser processing to modify aluminum and copper current collectors, generating hierarchical surface structures composed of micro- and nanoscale irregularities. This treatment significantly enhanced surface wettability, as evidenced by a reduction in contact angle from 50 to 34°. The resulting morphology improved both the adhesion strength and the electron transfer efficiency between the current collector and the active material. Consequently, the graphite-based anode exhibited enhanced high-rate capability, maintaining performance up to 4C, and demonstrated extended cycle life exceeding 600 cycles.^39^

Romoli et al. utilized laser pulse treatment to generate micrometer-scale craters—up to 7 μm in depth, on copper and aluminum current collectors. This process significantly increased the surface roughness of the foils. As a result, the adhesion strength between the active material layer and the current collector, assessed via peel-off tests, was enhanced by approximately 30%, indicating a notable improvement in adhesion strength.^41^

Circular craters with an average diameter of approximately 35 μm and a depth of around 6 μm were fabricated on the surface of an aluminum current collector using nanosecond fiber laser pulses. Subsequent electrochemical testing of LiFePO_4_ (LFP) electrodes revealed enhanced wettability and improved specific capacity. These improvements were attributed to the strengthened interfacial contact between the current collector and the slurry, as well as more efficient electron transport to the active material. The performance enhancement was particularly pronounced under high discharge current densities.^42^

Femtosecond laser pulses (FLP) have also been employed to ablate small through-holes, approximately 30 μm in diameter, in aluminum current collectors. This laser-induced perforation resulted in significantly enhanced electrochemical performance, with improved C-rate capabilities up to 60C and extended cycling stability exceeding 1100 cycles at 1C. These enhancements were attributed to reduced internal resistance and an increased apparent lithium-ion diffusion coefficient, facilitating more efficient charge transport during high-rate operation.^43^

From the many different methods of structuring the current collectors for Li-ion batteries, laser treatment seems to be one of the most beneficial and easily scalable. This paper presents the results of femtosecond laser modification of the surface of copper current collectors aimed at extending the lifetime of the silicon anode. Two types of copper foils were utilized to compare the surface patterning effect on the improved battery stability.

## 2 Experimental Section

### 2.1 Current Collector Preparation

To prepare the current collectors, copper foils with 18 and 100 μm thicknesses were used, signed as samples C_1 and C_2, respectively. Laser micromachining technology using ultrashort pulse technology was used to obtain a developed surface on the copper current collector. Yb:KGW femtosecond laser (λ = 1030 nm, τ = 270 fs, *P* = 4 W, Pharos 04–500- PP, Light Conversion) together with a galvoscanner (SCANCube III 14, ScanLab, Germany) based microfabrication setup (FemtoLAB, Altechna R&D, Lithuania) was employed to C_1 and C_2 sample structurization. The laser emitted linearly polarized light pulses with a duration of 270 fs with an adjustable repetition frequency. The laser beam was expanded up to 14 mm, scanned with a galvanometer scanner, and focused with a telecentric f-theta lens with a focal length of 72 mm. The beam spot size on the sample surface was ca. 12 μm at the 1/*e* ^2^ intensity level. The laser energy density had a Gaussian distribution. The copper foil was placed on an X,Y-coordinate table with computer control (8MT167, Standa, Lithuania). The structuring process was performed for all experiments in an ordinary atmosphere at room temperature, as well as with a normal incidence of the laser beam. The micromachining conditions are summarized in Table 1.

**Table 1:** Femtosecond Laser Parameters Applied for Copper Foils Structurization

material	C_1	C_2
wavelength λ (nm)	1030	
laser medium	Yb:KGW	
pulse width (fs)	270	
repetition frequency (kHz)	500	300
scanning speed *V* (m s^–1^)	1	0.15
laser fluence *F* (J cm^–2^)	0.54	1.98
step (μm)	5	15

### 2.2 Scanning Electron Microscopy

SEM imaging of the modified current collectors was performed by FESEM Helios NanoLab 650 (FEI, Hillsboro, Oregon) using an ETD detector with a Secondary Electron (SE) imaging mode microscope (25 kV acceleration voltage, 7.8 mm working distance) for C_1 samples (Center of micro- and nanotechnology UR) and a Carl Zeiss Merlin microscope (3 kV acceleration voltage, 2.8 mm working distance) for C_2 samples (University of Warsaw).

### 2.3 Electrochemistry

#### 2.3.1 Electrode Preparation and Characterization

A foil roughness test was conducted using an OLYMPUS LEXT OLS5100 laser confocal profilometer with a 50× MPLAPON50xLEXT objective (laser wavelength = 405 nm). Measurements were carried out to determine differences in surface roughness parameters between laser-treated and untreated regions.

To examine the application of a laser-modified copper layer as a current collector in lithium-ion batteries, silicon nanoparticle SiNP was chosen as an electroactive species. The silicon nanopowder (Sigma-Aldrich-633097) particle size was below 100 nm as stated by the manufacturer’s COA. The SEM observation (Figure S1) confirmed that all of the visible SiNPs have a diameter below 300 nm, and a majority of them are even lower than 100 nm. The LPSA (laser particle size analysis, Bettersizer S3 Plus) confirmed that the solution of the SiNP consists mainly of 30–200 nm isolated particles (up to 35% total no.), small aggregates of few NPs with a total diameter of up to 1 μm (40% total no.), and small amounts of larger aggregates up to 6 μm (Figure S2). The number of large aggregates in the slurry of the active material should be even lower due to higher shearing forces resulting from the mixing of high-viscosity fluid.

Slurry preparation started by grounding silicon nanoparticles with Vulcan XC72R conductive carbon (Cabot) in an agate mortar for 20 min. Then, a 3.5% carboxymethyl cellulose (CMC, Sigma-Aldrich) solution in deionized water was added to the so-prepared powder mix. The mass ratios of Si/Vulcan/CMC in the obtained slurry were 60:25:15. The resulting mixture was further homogenized for 24 h on a magnetic stirrer. After the homogenization process, the as-obtained slurry was coated by Elcometer 3545 through the total organic carbon (TOC) automatic film applicator onto standard copper foil (9 μm thickness, labeled as “untreated,” Shandong Gelon Lib Co., Ltd.) and C_1, C_2 samples. The obtained layer was left to dry in air for 1 h and then placed into a vacuum oven for further drying at 120 °C.

Lastly, round electrodes, 9 mm in diameter, were cut from the foils, pressed under a hydraulic press at 3000 kg for 15 s, thoroughly weighed, and transferred into a vacuum oven for drying at 120 °C. Prepared electrodes were then immediately transferred to an argon-filled glovebox (MBraun, H_2_O/O_2_ < 1 ppm) for cell preparation.

The mass loadings of the prepared electrodes were 0.93 ± 0.02, 0.96 ± 0.05, and 1.52 ± 0.02 mg cm^–1^ for the standard, C-1, and C_2 samples, respectively. The mass loading refers to the amount of silicon active material in the electrode. The noticeably greater loading for the C_2 electrodes resulted from additional mass stored inside deep grooves occurring at the foil surface. Typical thickness of the electrodes’ active layer above the textured surface (over the grooves) after calendering ranged from 10 to 20 μm (measured by MC-CT200 magneto-induction layer gauge).

#### 2.3.2 Cell Manufacturing

The silicon electrodes were placed in three-electrode Swagelok cells, containing a Si-based layer as the working electrode and metallic lithium (Sigma-Aldrich) as both the counter and the reference electrodes. Working and counter electrodes were separated by a Celgard 2325 separator, soaked in 1 M LiPF_6_ solution in ethylene carbonate:dimethyl carbonate (EC:DMC) 1:1 + 5% fluoroethylene carbonate (FEC) and 3% vinylidene carbonate (VC) acting as an electrolyte. The FEC and VC addition was used due to its well-known properties of stabilizing the SEI layer formed at the Si-based electrode’s surface.

#### 2.3.3 Electrochemical Evaluation

The cells containing silicon electrodes were subject to galvanostatic charge/discharge cycling on a multibattery tester, ATLAS 0961 (Sollich). The cells were charged/discharged between 0.01 and 1.50 V (vs Li^+^/Li^0^, standard foil) or 0.01 and 1.20 V (vs Li^+^/Li^0^, modified foils) for 50 cycles, at 0.1 C current rate (where C corresponds to 3590 mA g^–1^, related to the mass of silicon). The electrochemically active surface area of the collectors was estimated by electrochemical impedance spectroscopy (EIS) (10 mV RMS amplitude) technique in a 3-electrode setup in a 10% w/w sulfuric acid, glass fiber separator, and the Hg/HgSO_4_ reference electrode. The EIS was performed by Solartron 1260 impedance/gain phase analyzer coupled with Solartron 1278 potentiostat/galvanostat.

## 3 Results and Discussion

### 3.1 Current Collector Morphology

The images of the treated surfaces of C_1 and C_2 samples are presented in Figure 1. The micromachining process regimes of copper foils are presented in Table 1. The parameters for C_1 structuring were mild and resulted in grooves creation with a spatial periodicity of about 700–800 nm. They have a clear predominant orientation perpendicular to the laser polarization. This pattern is related to the formation of LIPSS (laser-induced periodic surface structures). The mechanism of its formation is explained by the complex interference phenomenon between the generated plasmon wave on the surface and the impinging light.^44–47^ This effect, in turn, causes a spatial modulation of the light intensity on the surface of the material sample. This is accompanied by a spatial modulation of the temperature field, which eventually leads to this specific engraving.^48^ It was established that LIPSS on copper can be created using a laser flux close to the ablation threshold −0.41 J cm^–2^,^49^ which is consistent with our experiments (Figure 1D). One can observe a pattern of LIPSS for the C_1 sample (Figure 1A–C), whereas on the C_2 surface, evenly distributed grooves can be observed (Figure 1D).

**Figure 1:**
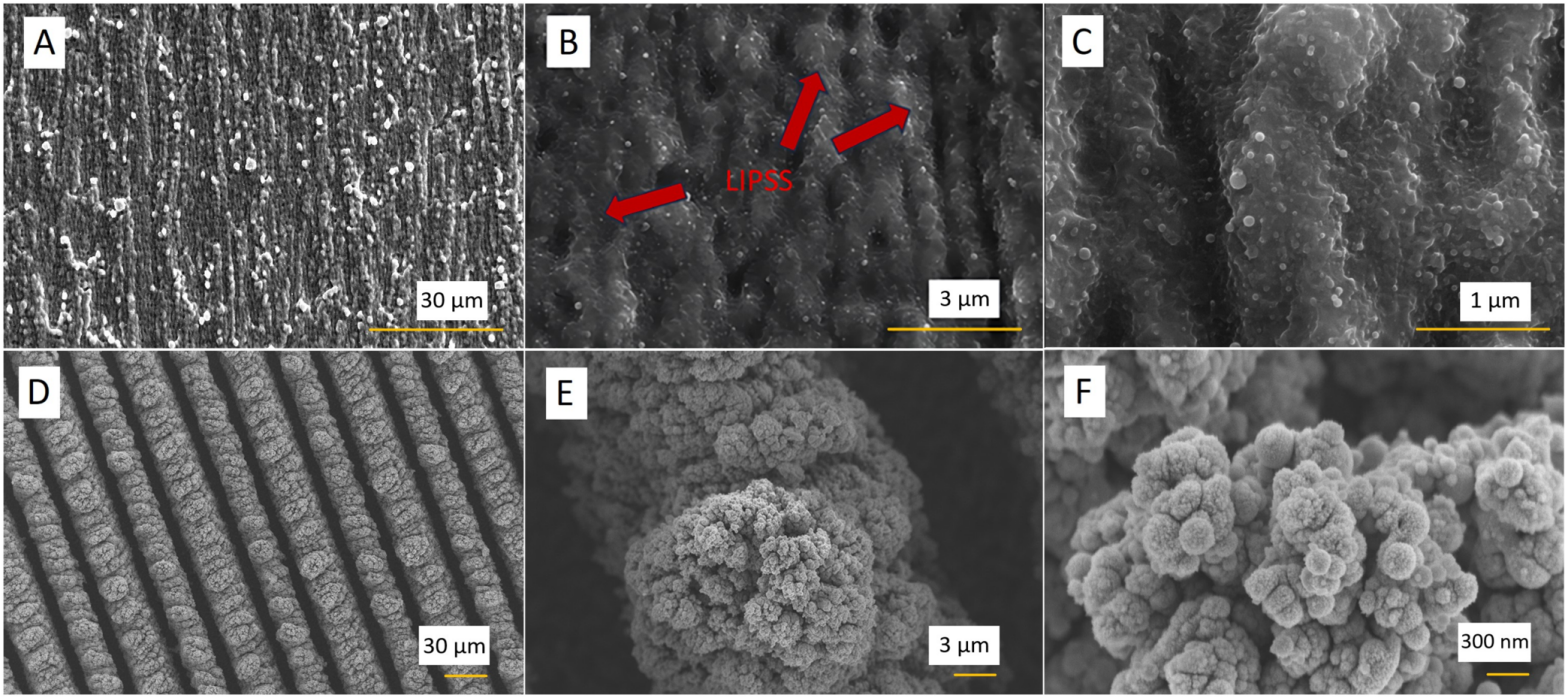
SEM images of the laser-treated sample C_1 in different magnifications (A–C) and C_2 sample in different magnifications (D–F).

In relation to sample C_2, the treatment by the femtosecond laser caused the Cu particles to evaporate and redeposit on the edges of the groove. These clusters of copper particles form a rough surface. Figure 1E,F shows that on the outer edges of the grooves a sphere-like particles (cone structures) covered with smaller structures “reminiscent of cauliflower” were formed. The cone structures are aligned with the direction of the scanning laser, forming rows, which can be seen in Figure 1D. The individual grains of cones are of 100–300 nm size and form a very porous structure. As can be seen from Table 1, the conditions for the formation of these structures are a higher energy density, a significant excess of the ablation threshold, and a larger number of pulses at a lower laser beam scanning speed. This regime leads to the accumulation of heat in the zone of influence and the superposition of complex hydrodynamic processes. This rather fast process makes it possible to manufacture samples with a triple hierarchical surface structure: scheme shallow, parallel, large microgrooves, a finer texture of grooves with a submicrometer period, and random decoration of nanoparticles.^50,51^ A combination of the deep grooves and porous structure that could withstand some plastic deformation seems to be beneficial for intended utilization as a silicon electrode support. As can be seen, the unique energy, time, and spectral modes of laser operation, as well as surface scanning modes, allow a multivariate structuring process.

The SEM images (Figure 1) suggest that sample surfaces differ significantly in roughness. The analyzed roughness parameters were as follows: *S*_a_—the arithmetical mean height and *S*_dr_—the developed interfacial area ratio (Table S1). Roughness analysis of the samples showed values of 0.239, 0.121, and 9.308 μm for untreated foil, C_1, and C_2 samples, respectively. The surface of the copper for the C_1 sample was partially melted, and thus the overall roughness was reduced; however, randomly distributed, high/low spots were formed. Despite the lower overall roughness of the foil, these spots may provide additional “anchoring” points for the active layers, promoting their adhesion.

These results are reasonable because the LIPSS structure is smooth, whereas grooves have spatial (3D) morphology. Profilometry results obtained for the C_1 sample (Figure S3 and Table S1) revealed a decrease in the surface roughness after laser treatment, whereas for C_2, one can observe increasing roughness due to laser structurization. The results are in good correlation with the SEM observations.

The approximated electrochemically active surface area (ECSA) of the modified current collectors was calculated by electrochemical impedance spectroscopy (EIS) as other direct surface estimation techniques such as N_2_ adsorption/desorption have insufficient sensitivity. The electrochemical response was fitted with an R_1_-L_1_-(R_2_)­(CPE_1_)-(R_3_)­(CPE_2_) equivalent circuit. The R_1_ and L_1_ originate from cable/connection resistance and inductance. The first semicircle is due to the response coming from current collectors and other cell elements used during the measurement, while the second one comes from the double-layer capacity of the measured copper foils (Figures S4 and S5). Constant phase element was used in the equivalent circuit due to differences in the distance between the measured electrode and the reference electrode. The CPE “*Q*” factor determined by eq 1, which is proportional to the double-layer capacity and by consequence to the electrode surface area, is presented in Table 2.

1 $$Z_{\mathrm{CPE}}=\frac{1}{{\left(i\omega \right)}^{\alpha }Q}$$

**Table 2:** Double-Layer CPE “*Q*” Factor and Relative Surface Area Obtained by EIS Measurement

sample	*Q* factor	relative surface area (%)
standard foil	0.000837 ± 0.000013	100
C_1	0.001015 ± 0.000023	122
C_2	0.012423 ± 0.000983	1484

The surface area for C_1 increased slightly by 22% in comparison to the pristine foil. The largest difference is visible for the C_2 sample, whose capacity is over 14 times higher than that of the flat foil. The results are in good agreement with SEM observation, where the C_1 foil surface features are rather smooth, leading to a small surface area change, while deep grooves and spongy-like structures on the C_2 sample are clearly contributing to an increase in the current collector surface area.

### 3.2 Electrochemical Performance

Charge/discharge curves of silicon electrodes with a standard current collector are presented in Figure 2A. The shape of the curves corresponds to typical silicon behavior in lithium-ion systems. The shape of the potential curve during the first lithiation (plateau at ca. 0.1 V vs Li^+^/Li^0^) is typical of the two-phase process. In the case of silicon lithiation, it does not represent two thermodynamically stable phases but rather a kinetic limitation of lithium insertion into the silicon lattice. The subsequent lithiation and delithiation show a typical single-phase process shape due to minor changes in the silicon lattice induced by amorphization and lithium trapping. In the first cycle, cells delivered 2267.3 ± 185.0/1645.5 ± 146.1 mAh g^–1^ during discharge and charge processes, respectively. The specific capacity of the material quickly dropped, reaching 366.2 ± 23.8/358.1 ± 22.1 mAh g^–1^ after 50 discharge/charge cycles, respectively (Figure 2B).

**Figure 2:**
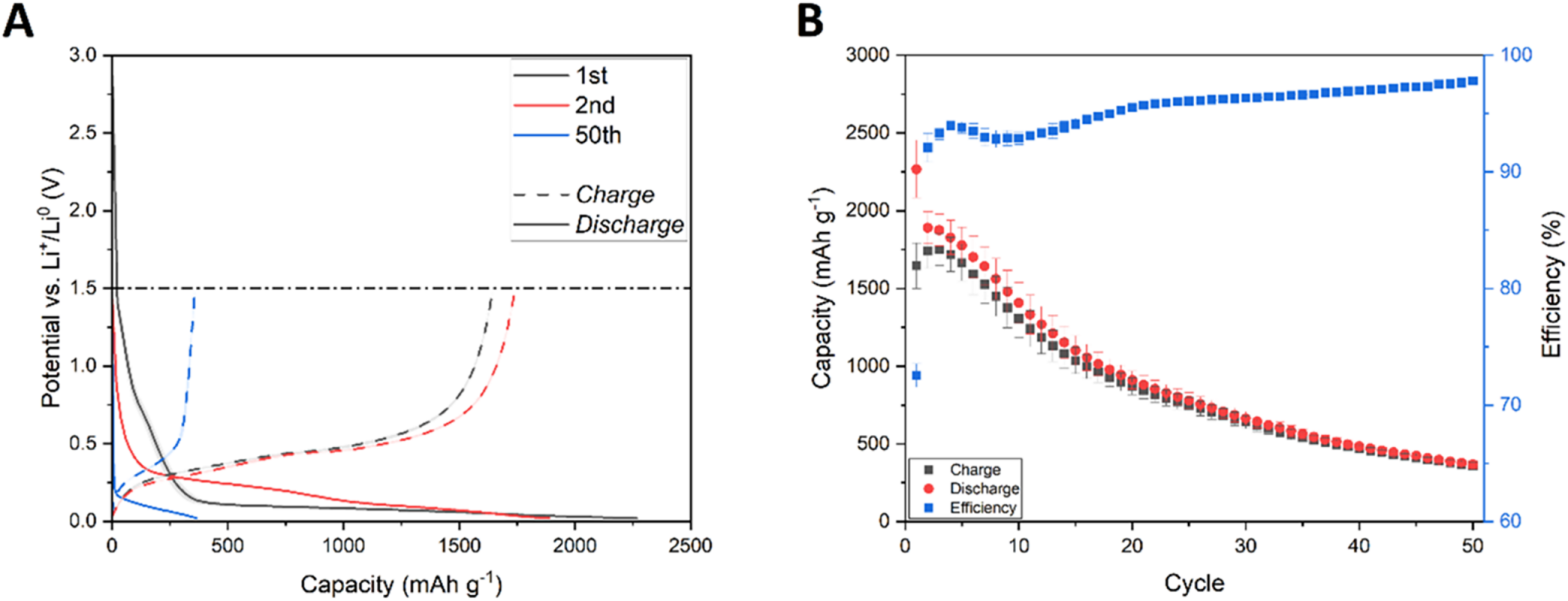
Charge and discharge curves (A) and cyclability (B) of silicon electrodes containing a standard copper current collector. Blurred areas and error bars correspond to the standard deviation of the arithmetic mean acquired from different electrochemical cells’ performance.

The retained capacity after 50 discharge/charge cycles was evaluated to be 16.4 ± 2.4/22.1 ± 3.3% for the discharge and charge processes, respectively. Such behavior in Li-ion cells is typical for pristine silicon, which electrochemically reacts with lithium through an alloying mechanism, resulting in phase transformations and large volume changes (up to 300%) throughout the discharge and charge processes. Those volume changes inevitably cause mechanical degradation of the electrode, which includes pulverization and separation of the material from the current collector.

A different behavior was observed for silicon powder coated onto modified copper current collectors. Figure 3A shows the discharge and charge curves of silicon electrodes with a modified C_2 current collector. One can observe that an additional signal appeared, at ca. 1.5 V (vs Li^+^/Li^0^), during the first discharge of the cell. The process is no longer visible in the next cycles, suggesting an irreversible reaction, which can be attributed to a reduction of copper oxides that were formed at the surface of the current collector during laser ablation. It was reported that the copper oxides undergo a multistep electrochemical reaction, associated with the creation of a Cu_1–*x*_IICu_*x*_IO_1–*x*/2_ intermediate, a Cu_2_O phase, and further reduction to Cu and Li_2_O.^52^ The CuO reduction occurred at 0.99 and 0.74 V vs Li^+^/Li^0^. Other compounds, Cu_2_O and Cu­(OH)_2_, were reported to be easily reduced at ca. 1.4 and 1.2 V vs Li^+^/Li^0^, respectively.^53^ The precise value was highly dependent on the morphology and electrolyte composition; thus, we believe that in the case of the C_2 sample, it is shifted toward higher potential. The copper­(II) hydroxide or copper­(I) oxide can be easily formed at the highly expanded copper surface after contact with a water-based slurry. A similar flash oxidation of copper leading to Cu­(I) compounds formation was observed after the preparation of a 3D current collector by electrodeposition of spongy copper.^21^ C_1 and unmodified electrodes did not show the distinct 1.5 V plateau due to much lower (or absence of) copper oxidation.

**Figure 3:**
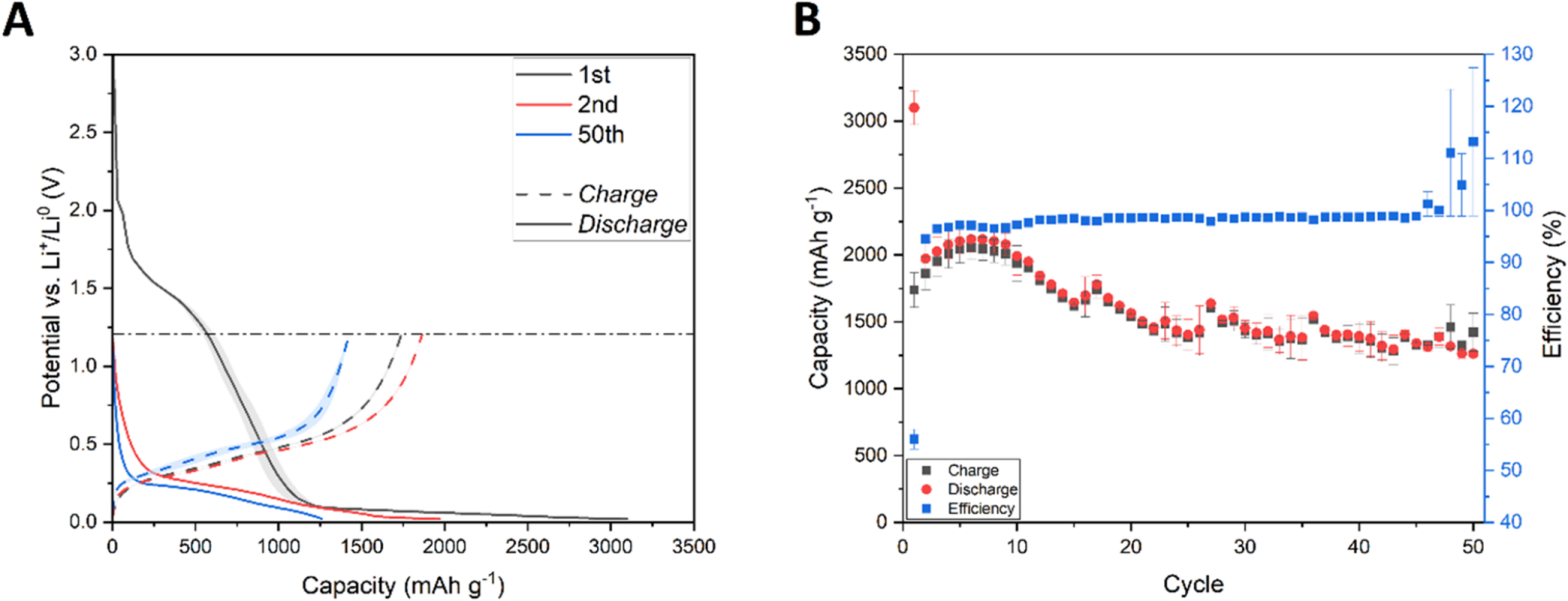
Charge and discharge curves (A) and cyclability (B) of silicon electrodes containing a modified C_2 copper current collector. Blurred areas and error bars correspond to the standard deviation of the arithmetic mean acquired from different electrochemical cells’ performance.

At the first discharge–charge cycle, the cells delivered 3100.4 ± 126.6/1738.4 ± 129.6 mAh g^–1^ during the discharge and charge processes, respectively. After 50 cycles, the cells retained 40.8 ± 2.7/81.7 ± 2.1% of their initial discharge/charge capacity, reaching 1261.0 ± 113.2/1422.3 ± 33.2 mAh g^–1^ during the 50th discharge and charge processes, respectively (Figure 3B).

The electrodes made from C_2 current collectors showed increased charge/discharge capacities and higher cyclability than the electrodes containing standard copper foil. This is the result of active material residing inside the etched rows in the copper current collector. The typical groove is over 7 μm wide, while the active material grain size is under 200 nm as seen by SEM images or mostly (>75%) below 1 μm in the case of agglomerates in the solution (confirmed by laser particle size analyzer, Figure S2). It is highly probable that some of the active slurry is stored inside the grooves that provide high constraints on the SiNPs. The difference in the mass loading between standard and C_2 electrodes suggests that 63% more active material was inserted into the grooves than was inserted into the standard copper foil. This material inserted into the groove is highly constrained; thus, its connection with the current collector is improved. As a consequence, the main reason for silicon electrode degradation (separation of active material from the copper collector or separation between particles inside the active layer) is hindered. On the other hand, porous copper morphology at the groove slopes can provide some kind of buffer effect to the silicon volume changes, additionally improving the electrical contact. A cross-section SEM image of the noncalendered electrode (Figure 4) (and additional Supporting Figure S6A–C) confirmed that the deep grooves of the C_2 sample were completely filled with active mass, which greatly improved its electrochemical stability.

**Figure 4:**
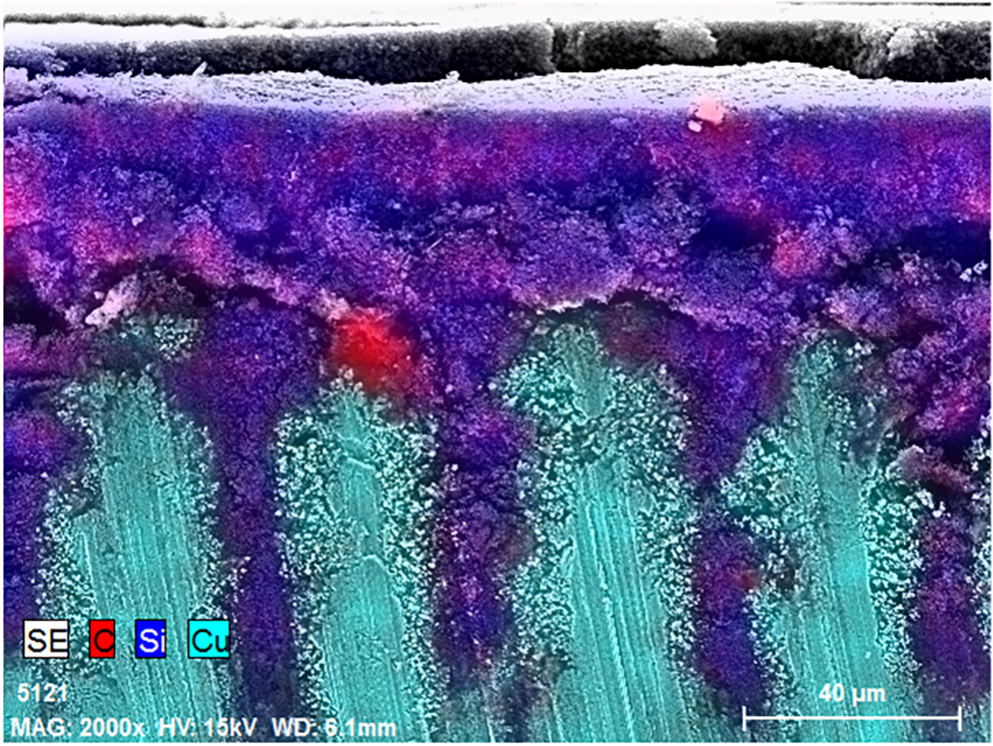
Cross-section SEM image of the C_2 electrode prior to calendering. Color overlap was based on EDS mapping.

In the initial cycles (1–6), the specific discharge capacity of the C_2 electrodes increased from 1738 to 2055 mAh g^–1^ due to activation phenomena originating from cracking or partial reduction of the SiO_2_ layer present at the SiNPs surface. It was proven that under 1.3 V vs Li^+^/Li^0^, the SiO_2_ layer is gradually reduced to Li_*x*_SiO_*y*_, which has improved electrical and ionic conductivity.^7,54^ Another explanation may be related to internal stress relaxation by full amorphization of microcrystalline domains inside SiNPs, microcracking, or plastic deformation of the active layer and current collector. The internal stress build-up inside SiNPs can generate an overpotential value of 60–125 mV GPa^–1,^ ^55^ and can slow down or totally block the reaction front before full silicon lithiation.^56^ Since this effect was mainly observed for the C_2 sample, we believe that the stress relaxation by current collector plastic deformation was the main reason for the observed phenomena. A similar explanation can be stated for the sharp capacity increase in cycles 16, 27, 36, and 44, in which we believe that stress build-up exceeded the matrix stiffness, which resulted in sudden deformation and, as a consequence, stress relaxation and capacity growth. Despite the stress-related phenomena, for most of the cycles, the modified surface of copper foil acts as a buffer zone for the volume expansion of silicon particles during galvanostatic cycling, thus preventing the electrode from cracking due to mechanical stress and keeping the silicon grains connected to the current collector. This results in a higher number of Si particles being electroactive during cycling, increasing their specific capacity and cyclability compared with the unmodified sample.

The Coulombic efficiency during C_2 electrode cycling quickly increased by over 98.5%, which proves the exceptional stability of the electrodes. Some fluctuations at cycles 46–50 are attributed to lithium dendrite formation at the surface of the counter electrode and are not related to the changes occurring in the working electrode.

A similar electrochemical response was observed for the electrodes containing a modified thin current collector (C_1 sample). Figure 5A shows discharge and charge curves of silicon coated onto modified, thin copper foil. During the first cycle, the cells delivered 3608.4 ± 285.2/2762.9 ± 211.2 mAh g^–1^ for the discharge and charge processes, respectively. Both of those capacity values are even higher than for the modified, thick current collector. Moreover, the high irreversible capacity during the discharge process, happening at ca. 1.5 V (vs Li^+^/Li^0^), is no longer present, suggesting less copper oxide presence and/or a more stable and/or thinner SEI layer created during the first discharge of the cells. The specific capacity of the cells dropped to 1504.1 ± 197.4/1486.6 ± 197.1 mAh g^–1^ after 50 discharge/charge cycles. The C_1 sample absolute capacity after 50 cycles is higher compared to the thick, modified electrodes (C_2), hovewer C_1 retained much less of its initial capacity. As a result, electrodes containing thin, modified copper foil retained 42.4 ± 8.8/54.7 ± 11.3% of their initial capacity after 50 consecutive charge/discharge cycles (Figure 5B).

**Figure 5:**
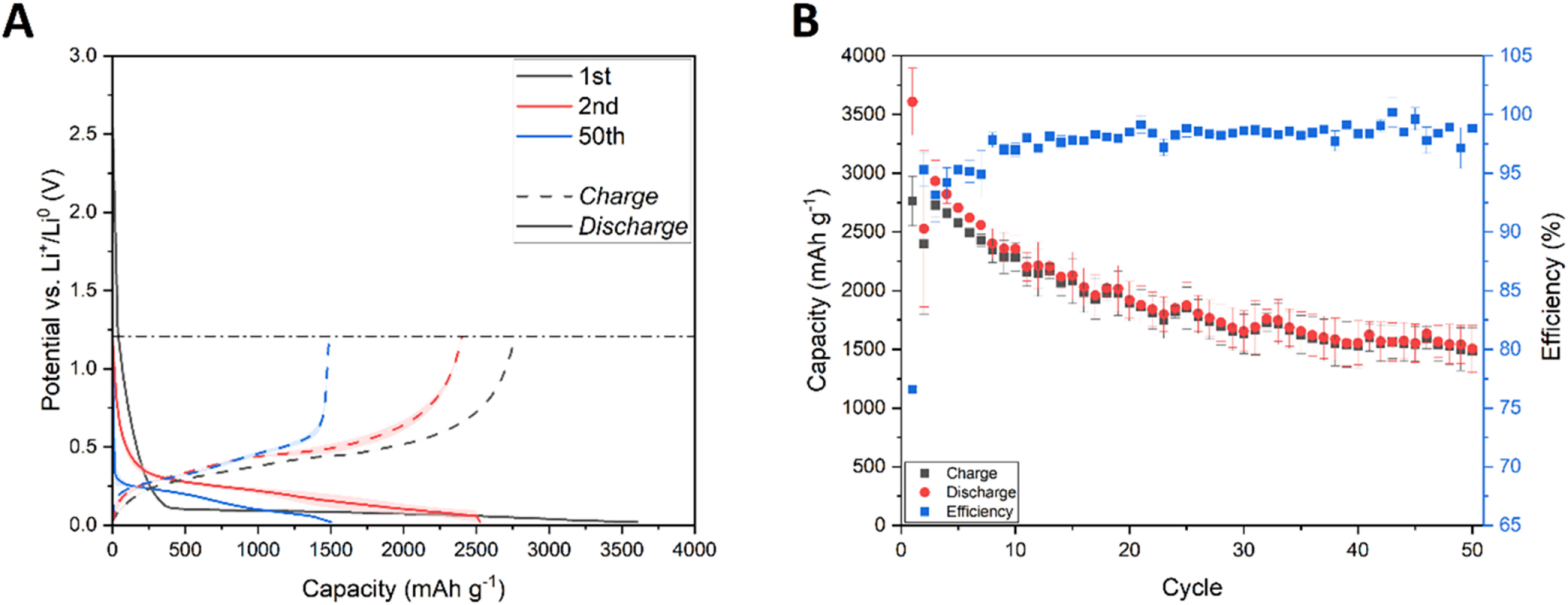
Charge and discharge curves (A) and cyclability (B) of silicon electrodes containing a modified C_1 copper current collector. Blurred areas and error bars correspond to the standard deviation of the arithmetic mean acquired from different electrochemical cells’ performance.

Some fluctuations of the specific capacity in subsequent cycles can be observed (Figure 5B) and can be attributed to a stress relaxation mechanism similar to the one described for C_2 samples. The amplitude of capacity fluctuation is however much smaller due to smaller critical stress values related to weaker constraining of the SiNPs by a thin current collector. A smaller maximum stress value between deformation is also evident as the inferior delithiation capacity retention for the C_1 (55%) samples compared with the C_2 (82%) ones.

Lack of capacity variation, accompanied by rapid capacity decay for the unmodified current collector, suggests that even small stress generation immediately resulted in active layer mechanical degradation and delamination rather than matrix/current collector plastic deformation.

One can see that modification of the copper surface by laser etching resulted in improvement of the electrochemical performance of silicon nanoparticles in lithium-ion cells. Both modifications increased the specific capacity of silicon and its cyclability, which is a result of better electrical contact with the active material grains throughout prolonged cycling. Moreover, the Coulombic efficiencies of modified electrodes were also increased and retained a value between 98 and 100% throughout the experiments (Figures 2B, 3B, and 5B). Increased efficiency can be attributed to better mechanical stability of the electrodes, due to silicon grains being constrained inside the etched copper current collector, and a higher total adhesion force between the active layer and current collector. As the SiNPs are highly constrained (especially inside C_2 sample deep grooves), we must stress that the individual grain volume changes associated with silicon lithiation/delithiation are only marginally hindered. The main reason for the better stability of modified electrodes is related to the elastic deformation of the current collector (plastic deformation occurs after reaching the critical stress value as described earlier) and increased adhesion force between copper and the active layer (the total force is proportional to the contact surface area, which is greater for modified collectors).

Reducing the thickness of the modified copper foil enhanced the initial specific capacities further, which can be related to the reduced polarization of thin electrodes undergoing a current flow through their area. The comparison between all three copper current collectors is also presented in Figure 6 and Table 3.

**Figure 6:**
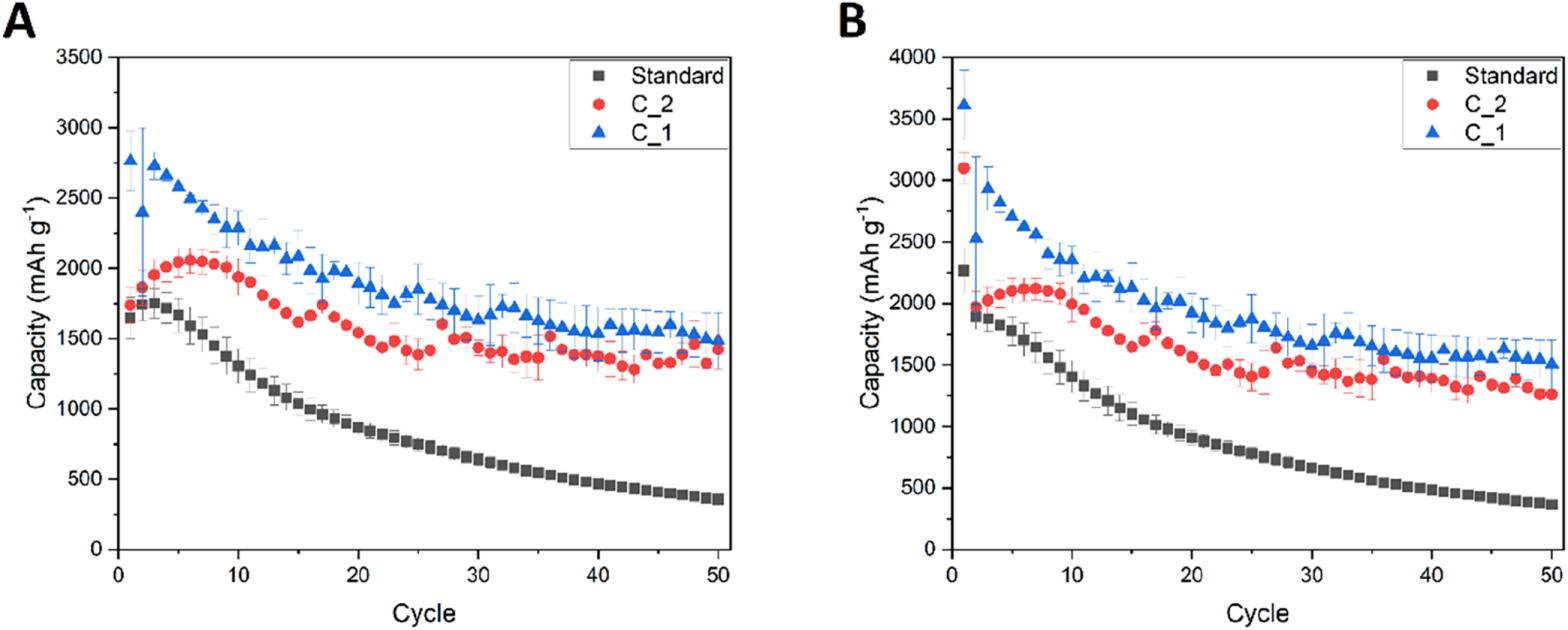
Charge (A) and discharge (B) specific capacities of unmodified and modified silicon electrodes; black squares, standard Cu foil; blue triangle, C_1; red circle, C_2. Error bars correspond to the standard deviation of the arithmetic mean acquired from different electrochemical cells’ performance.

**Table 3:** Summary of Chronopotentiometry Tests

current collector	1st discharge/charge capacity (mAh g^–1^)	2nd discharge/charge capacity (mAh g^–1^)	50th discharge/charge capacity (mAh g^–1^)	discharge/charge capacity retention (%)
unmodified	2267.3 ± 185.0/1645.5 ± 146.1	1890.5 ± 103.2/1741.7 ± 113.5	366.2 ± 23.8/358.1 ± 22.1	16.4 ± 2.4/22.1 ± 3.3
C_1	3608.4 ± 285.2/2762.9 ± 211.2	2526.4 ± 665.1/2398.6 ± 599.0	1504.1 ± 197.4/1486.6 ± 197.1	42.4 ± 8.8/54.7 ± 11.3
C_2	3100.4 ± 126.6/1738.4 ± 129.6	1970.7 ± 127.1/1862.5 ± 125.1	1261.0 ± 33.2/1422.3 ± 142.4	40.8 ± 2.7/81.7 ± 2.1

## 4 Conclusions

The applied femtosecond laser modification of the current collectors produced a rough surface with quasiperiodic lines that provided better mechanical stability during silicon volume changes. The effect was attributed to a larger contact area between Si grains/binder and copper surface, mechanical trapping of the slurry between deep grooves, and a more uniform charge distribution. The best sample retained 81.7% of the initial capacity after 50 cycles in comparison to only 22.1% for the flat copper foil. Electrodes with modified current collectors also showed better cycle efficiency, which can lead to lower lithium and electrolyte losses during cell operation. The presented modification technique is fast and scalable and can be used for any anode-active material, including commercial graphite/silicon blends. The presented method of copper structuring by laser ablation has wide application possibilities and can extend the life cycle of batteries by using variable-volume anode materials.

## Supplementary Material



## Notes

Additional experimental results, including SEM images of silicon active material; particle/agglomerate size analysis for used materials; laser confocal profilometer of used current collectors; additional electrochemical EIS graphs (PDF)
