# Supplementary material for: Femtosecond Laser Treatment of Copper Current Collectors and Their Application in Li-Ion Batteries
Source: ACS Appl Eng Mater. 2025 Sep 16;3(9):3228–38. doi: 10.1021/acsaenm.5c00589 (PMC12481476; doi:10.1021/acsaenm.5c00589)
Supplement: Supplementary file 1 [file em5c00589_si_001.pdf]

Supporting Information

Femtosecond Laser Treatment of Copper Current Collectors and Their Application in Li-Ion Batteries

Maciej Ratynski,[1] Michal Krajewski,[1*] Tomas Tamulevičius,[2] Yaroslav Vasyliovych Bobytskyy,[3] Joanna B. Kisała,[3] Piotr Krzeminski,[3] Bartosz Hamankiewicz[1] and Andrzej Czerwinski[1]*

[1]Faculty of Chemistry, University of Warsaw, Pasteura 1, 02-093 Warsaw, Poland

[2]Institute of Materials Science, Kaunas University of Technology, K. Baršausko St. 59, 51423, Kaunas, Lithuania

[3]Faculty of Science and Technology, University of Rzeszow, Pigonia 1, 35-310 Rzeszow, Poland

[*]corresponding authors:
mratynski@chem.uw.edu.pl
michal.krajewski@uw.edu.pl

Supporting Information description

Additional experimental results, including: SEM images of silicon active material, particle/agglomerate size analysis for used materials, laser confocal profilometer of used current collectors, and additional electrochemical EIS graphs.

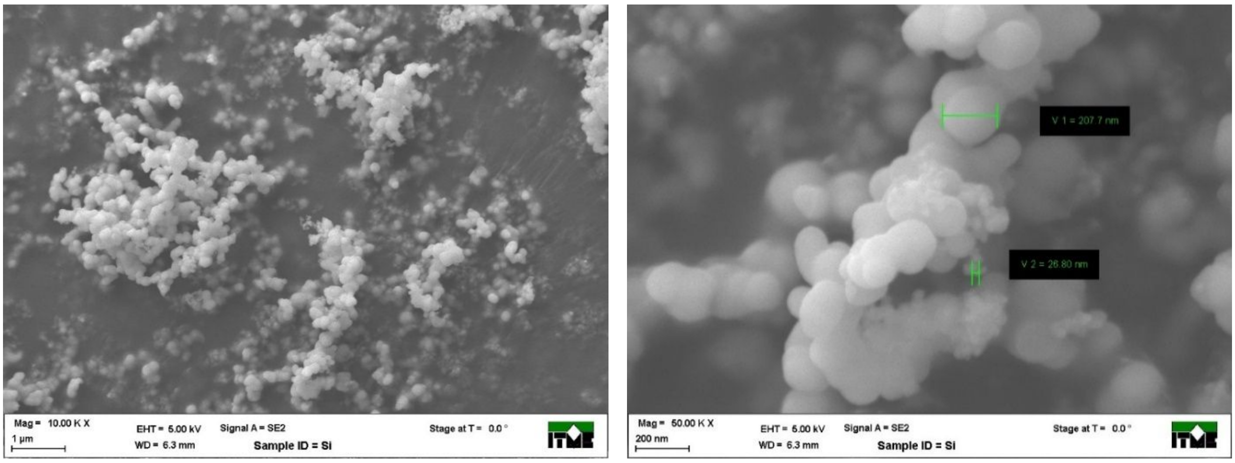

Figure S1. SEM images of pristine silicon powder presented at different magnification

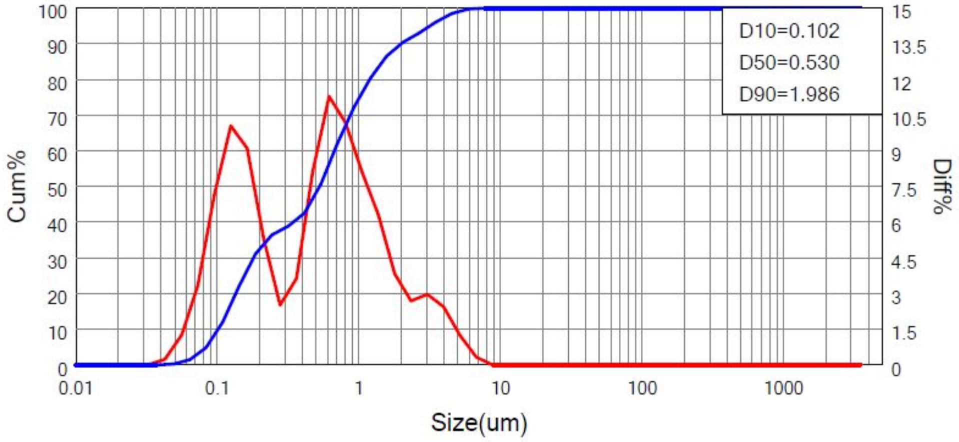

Diam um	Percent
0.020	0.00
0.050	0.30
0.100	9.32
0.200	32.79
0.500	47.90
1.000	74.76
2.000	90.09
5.000	98.98
10.00	100.00
20.00	100.00

Diam um	Diff%	Cum%	Diam um	Diff%	Cum%	Diam um	Diff%	Cum%
0.010-0.013	0.00	0.00	0.704-0.919	10.19	72.13	49.66-64.79	0.00	100.00
0.013-0.017	0.00	0.00	0.919-1.199	8.10	80.23	64.79-84.53	0.00	100.00
0.017-0.022	0.00	0.00	1.199-1.565	6.30	86.53	84.53-110.2	0.00	100.00
0.022-0.028	0.00	0.00	1.565-2.041	3.82	90.35	110.2-143.8	0.00	100.00
0.028-0.037	0.00	0.00	2.041-2.663	2.69	93.04	143.8-187.7	0.00	100.00
0.037-0.049	0.23	0.23	2.663-3.475	2.96	96.00	187.7-244.9	0.00	100.00
0.049-0.064	1.26	1.49	3.475-4.534	2.44	98.44	244.9-319.5	0.00	100.00
0.064-0.083	3.34	4.83	4.534-5.916	1.23	99.67	319.5-416.9	0.00	100.00
0.083-0.109	7.11	11.94	5.916-7.718	0.33	100.00	416.9-543.9	0.00	100.00
0.109-0.142	10.04	21.98	7.718-10.07	0.00	100.00	543.9-709.6	0.00	100.00
0.142-0.186	9.11	31.09	10.07-13.13	0.00	100.00	709.6-925.8	0.00	100.00
0.186-0.243	5.29	36.38	13.13-17.14	0.00	100.00	925.8-1207	0.00	100.00
0.243-0.317	2.51	38.89	17.14-22.36	0.00	100.00	1207-1576	0.00	100.00
0.317-0.414	3.63	42.52	22.36-29.17	0.00	100.00	1576-2056	0.00	100.00
0.414-0.540	8.14	50.66	29.17-38.06	0.00	100.00	2056-2682	0.00	100.00
0.540-0.704	11.28	61.94	38.06-49.66	0.00	100.00	2682-3500	0.00	100.00

Figure S2. The LPSA (laser particle size analysis) of the pristine silicone powder. Measured on Bettersizer S3 Plus Particle Size Analysis.

The measurement was conducted using OLYMPUS LEXT OLS5100 laser confocal profilometer with 50x MPLAPON50xLEXT objective (laser wavelength= 405nm). Specified areas contain both laser-treated (darker area) and un-treated (lighter area) regions. Measurements were carried out to determine differences in surface roughness parameters between laser-treated and un-treated regions.

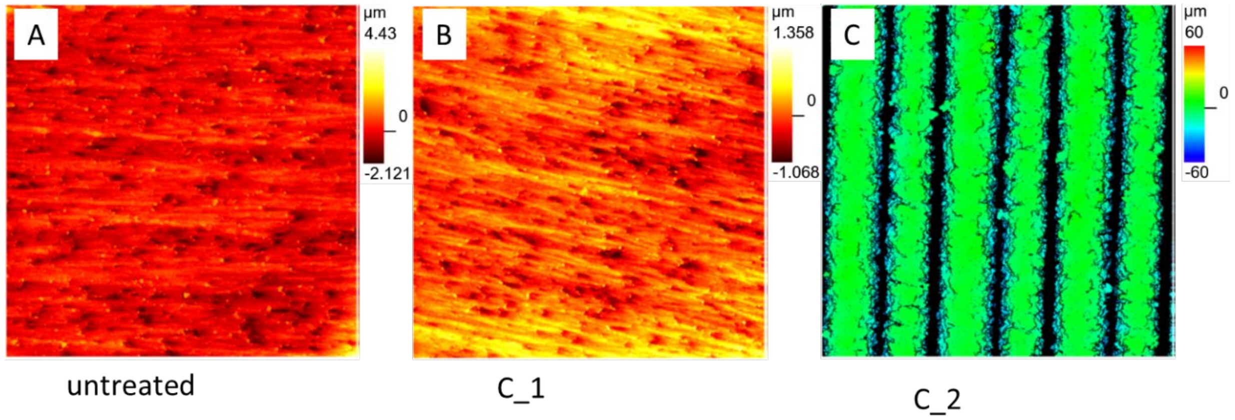

Figure S3. Images show 3D confocal height-scans of: A) un-treated foil and B) C_1 and C) C_2. Similar measurements and filters were used for all areas.

Table S1. Conducted meausrements using OLYMPUS LEXT OLS5100 3D confocal laser profilometer, where: S_a - Arithmetical mean height, S_{dr} - Developed interfacial area ratio

Specimen area	S_a (µm)	S_{dr} (%)
Un-treated	0,239	11,429
C_1	0,121	2,746
C_2	9.803	242.731

Conducted measurements provide enough data to define both areas: the laser- treated region has 50,6% smaller S_a parameter, which means it has much smoother surface comparing to un- treated region. Similarly the S_{dr} parameter in laser- treated zone is significantly smaller comparing to un-treated area, which means its' surface less developed (smaller effective area).

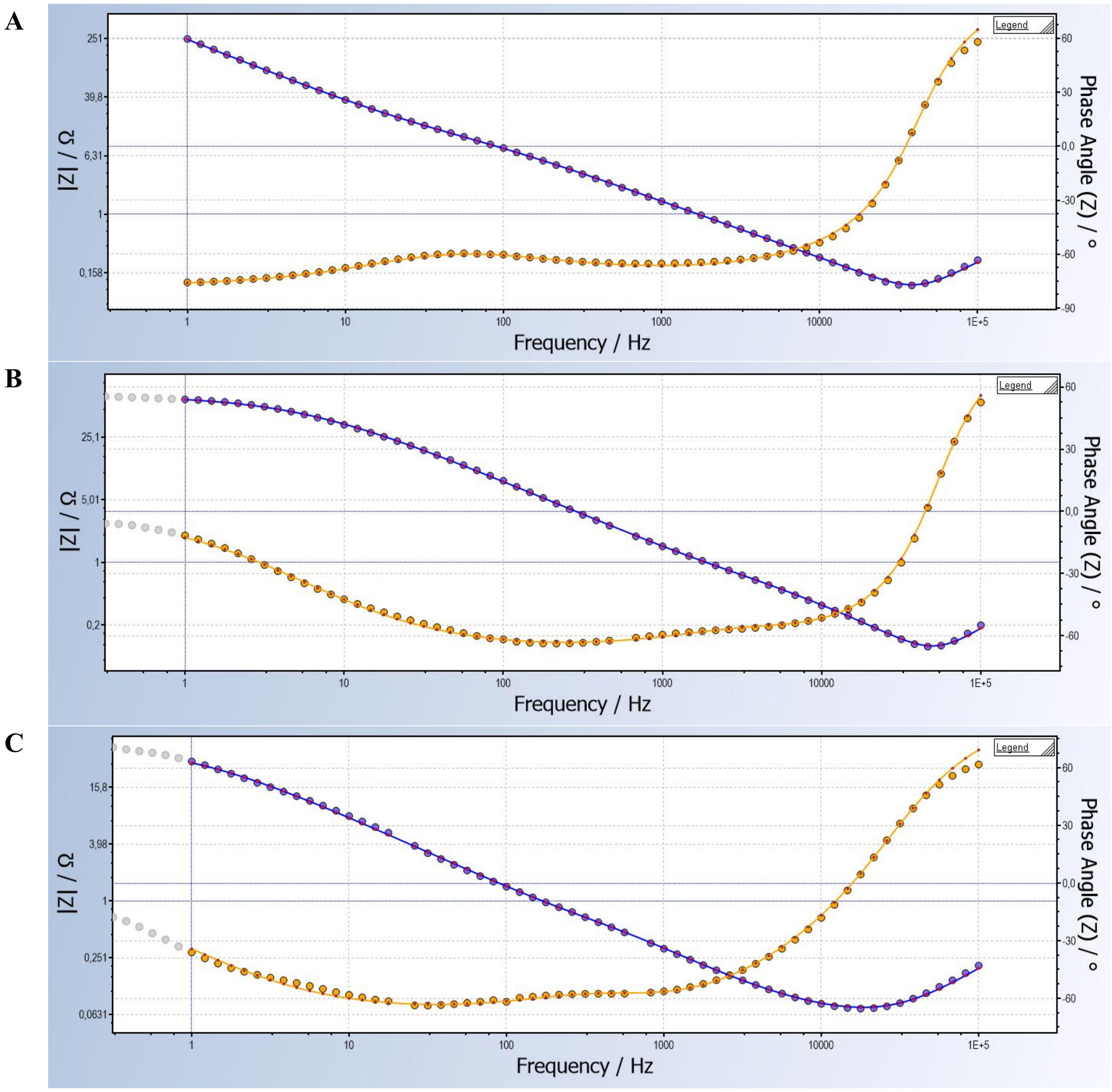

Figure S4. Electrochemical impedance spectroscopy data for standard copper foil (A), C_1 (B) and C_2 (C). Lines correspond to fitted equivalent circuit response.

A2

B2

C2

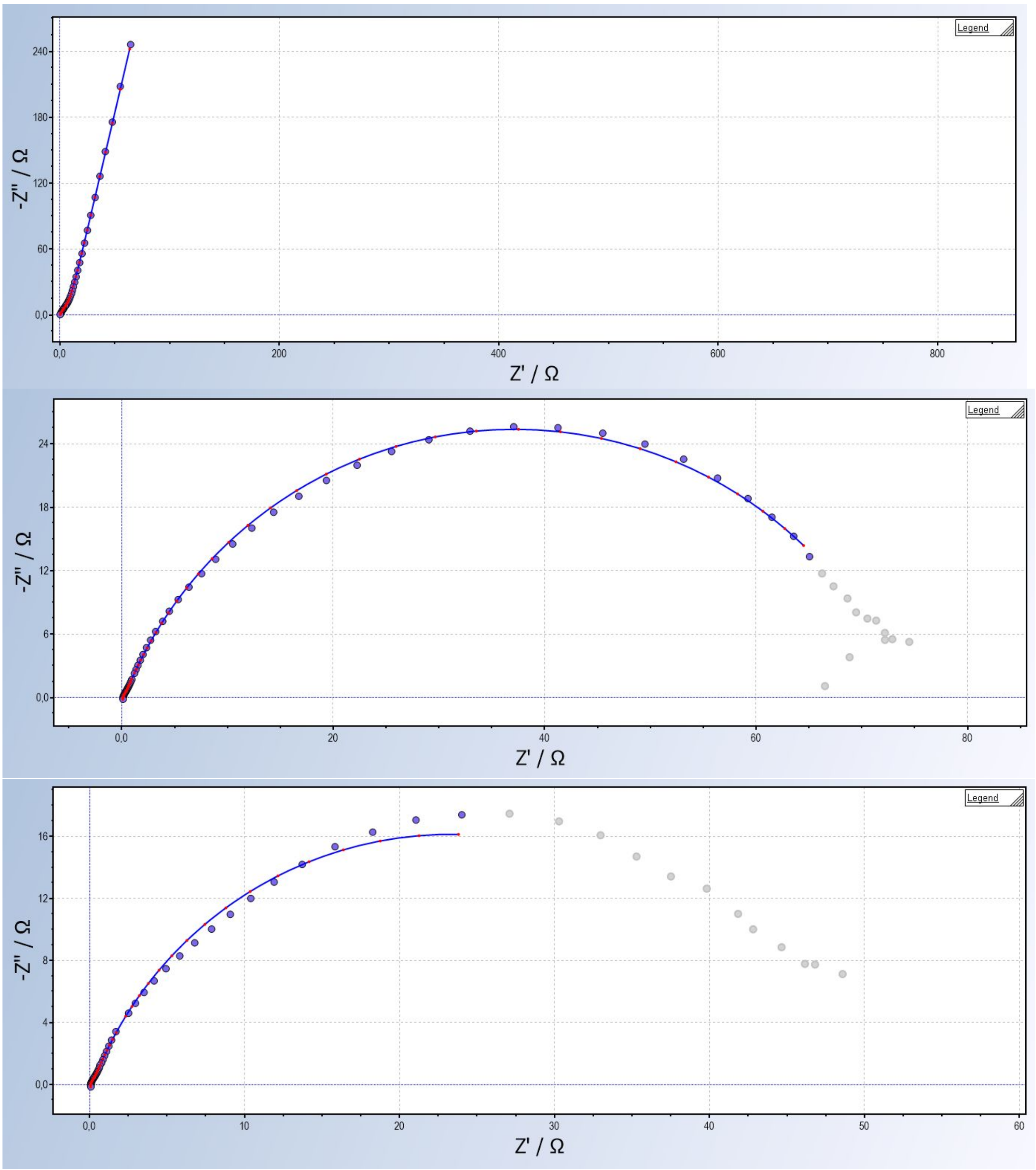

Figure S5. Electrochemical impedance spectroscopy data for standard copper foil (A2), C_1 (B2) and C_2 (C2). Lines correspond to fitted equivalent circuit response between $10^5$and 10^0 Hz.

A

B

C

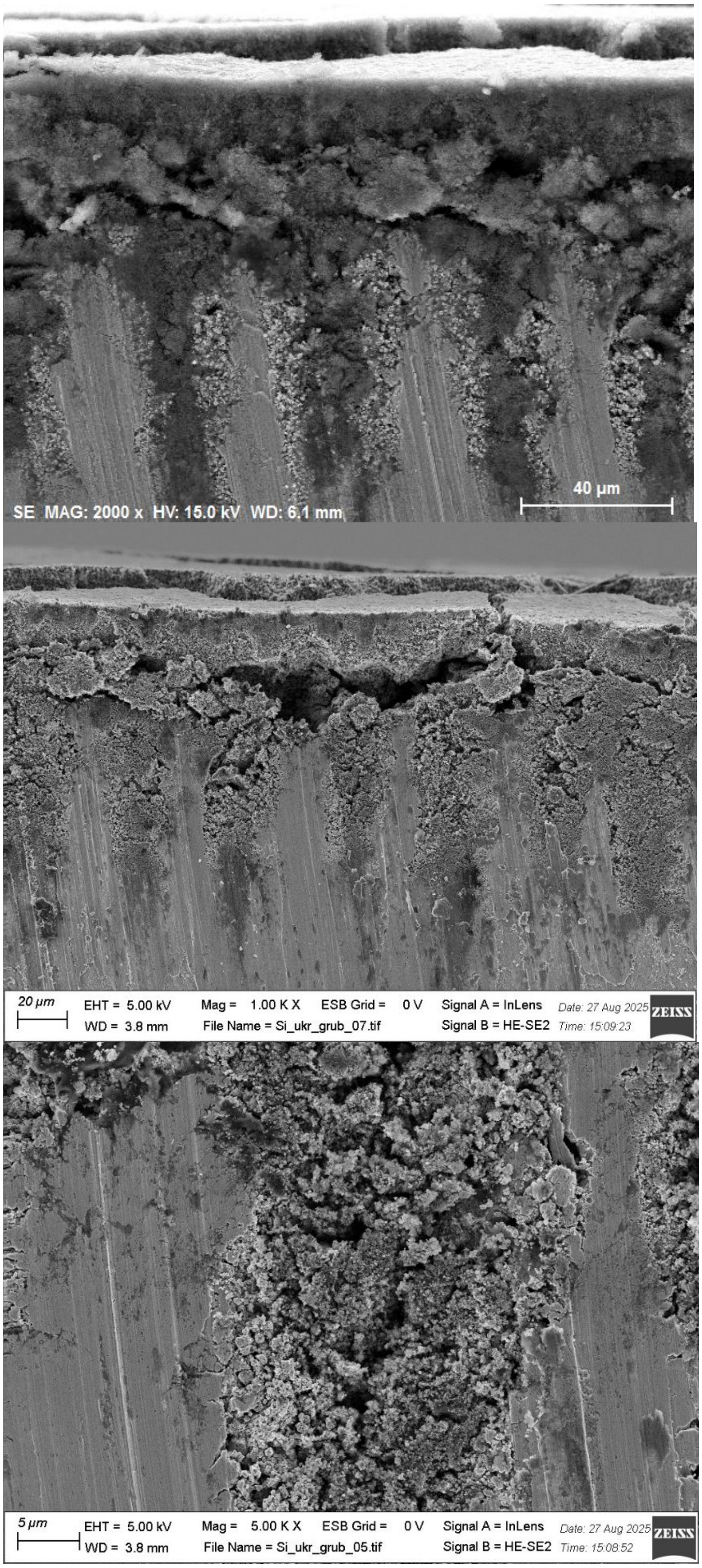

Figure S6. Cross-section SEM images (A,B,C) of C_2 electrode prior calendaring

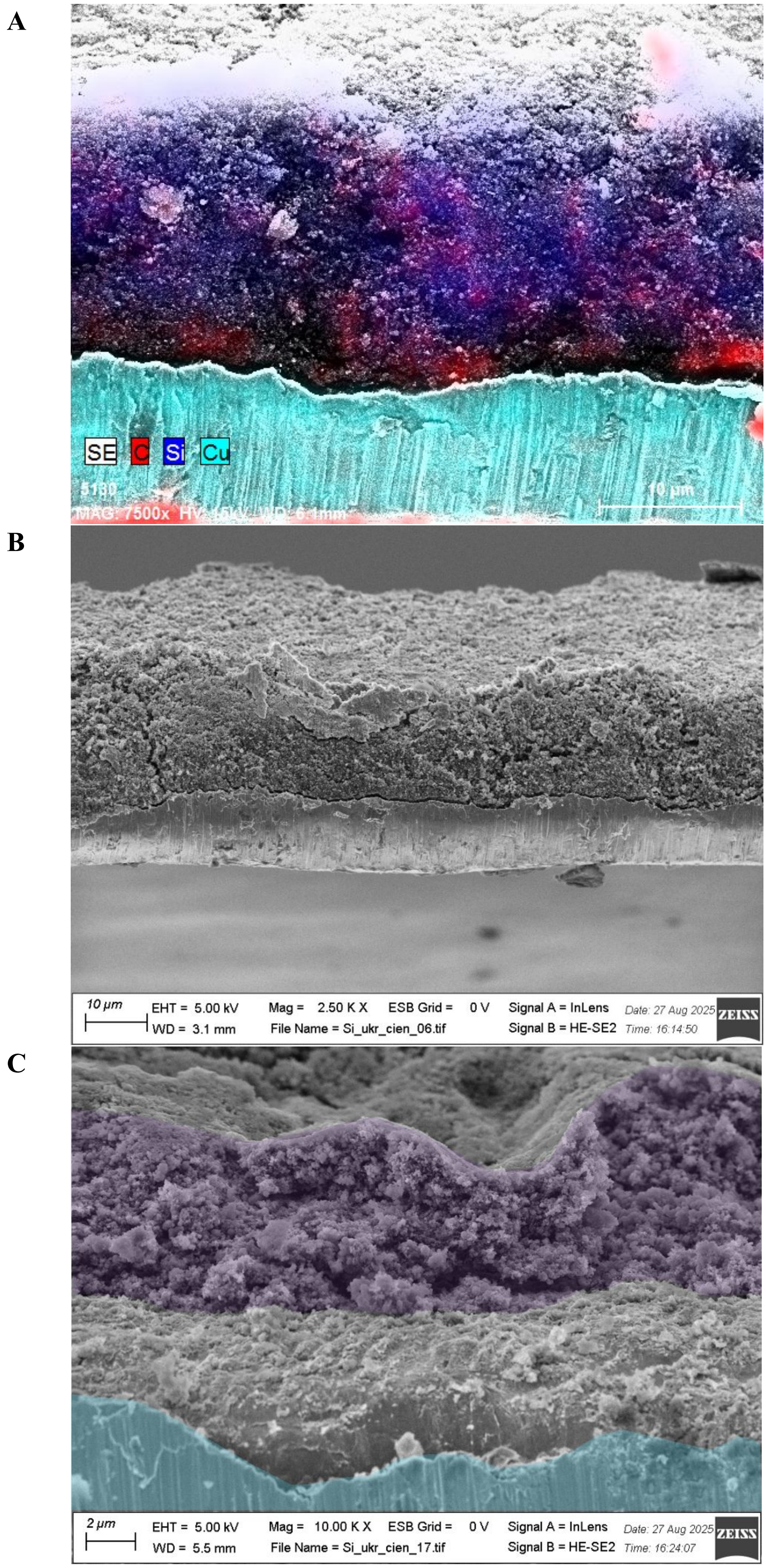

Figure S7. Cross-section SEM images of C_1 electrodes prior calendaring (A – clean cut with EDS overlay, B- clean cut region without EDS overlay, C- rough cut, with visible partial delamination of active layer near the edge - colour overlapping only for clarity of the image)
